# “Phoenix in Flight”: an unique fruit morphology ensures wind dispersal of seeds of the phoenix tree (*Firmiana simplex* (L.) W. Wight)

**DOI:** 10.1186/s12870-022-03494-z

**Published:** 2022-03-12

**Authors:** Shi-Rui Gan, Jun-Cheng Guo, Yun-Xiao Zhang, Xiao-Fan Wang, Lan-Jie Huang

**Affiliations:** 1 College of Life Sciences, Wuhan University, Wuhan, 430072 China; 2 University of Chinese Academy of Sciences, Beijing, 100049 China; 3 College of Life Sciences, Hubei University, Wuhan, 430062 China

**Keywords:** Curved surface, Flight performance, Fruit development, Spinning, Wind dispersal, *Firmiana*

## Abstract

**Background:**

Many seed plants produce winged diaspores that use wind to disperse their seeds. The morphology of these diaspores is directly related to the seed dispersal potential. The majority of winged diaspores have flat wings and only seeds; however, some angiosperms, such as *Firmiana* produce winged fruit with a different morphology, whose seed dispersal mechanisms are not yet fully understood. In this study, we observed the fruit development of *F. simplex* and determined the morphological characteristics of mature fruit and their effects on the flight performance of the fruit.

**Results:**

We found that the pericarp of *F. simplex* dehisced early and continued to unfold and expand during fruit development until ripening, finally formed a spoon-shaped wing with multiple alternate seeds on each edge. The wing caused mature fruit to spin stably during descent to provide a low terminal velocity, which was correlated with the wing loading and the distribution of seeds on the pericarp. When the curvature distribution of the pericarp surface substantially changed, the aerodynamic characteristics of fruit during descent altered, resulting in the inability of the fruit to spin.

**Conclusions:**

Our results suggest that the curved shape and alternate seed distribution are necessary for the winged diaspore of *F. simplex* to stabilize spinning during wind dispersal. These unique morphological characteristics are related to the early cracking of fruits during development, which may be an adaptation for the wind dispersal of seeds.

**Supplementary Information:**

The online version contains supplementary material available at 10.1186/s12870-022-03494-z.

## Background

Seed dispersal plays an important role in offspring survival and population establishment of seed plants [1, 2]. These species have evolved various biological or abiotic mechanisms to aid in the spread of seeds to appropriate areas [3, 4]. Among these mechanisms, wind dispersion is commonly utilized, with approximately 10% –30% of seed plants relying on wind to distance their seeds from the mother plant [5].

There are various flight modes of diaspores (dispersed unit) depending on the flight characteristics of seeds and the force applied during descent, such as floating, gliding, and spinning [6, 7]. Different flight appendages are generally required for the various flight modes [8–10]. Although there may be considerable variation in the structures of these flight appendages, they all serve to increase the dispersal distance of seeds by reducing the descent velocity after leaving the maternal plants and keeping them in the air for an appropriate duration [11–13]. Certain gymnosperm cones and angiosperm families with distant phylogenetic relationships, along with other plant groups, contain flight appendages that resemble a thin wing, which allow diaspores to spin steadily in the air after leaving the maternal plants [14, 15].

Winged diaspores that use spinning have strong positive correlations between the descent velocity and wing loading, similar to diaspores with other flight methods [6, 7, 16]. However, these winged diaspores require a complex aerodynamic mechanism to slow the seeds as they fall. The wings utilize both drag and lift forces to balance dispersal unit [7, 17, 18]. The lift force is mainly provided by the leading-edge vortex formed at the upper edge of the wing, which is common in animal flight [15, 19, 20]. This complex mechanism indicates that the morphology of the winged diaspore, especially the shape of the wing, is important role for the dispersal of seeds [14, 15, 21, 22]. Although many studies have discussed the relationship between the morphology of winged diaspores and their dispersal process, the majority are in relation to wings with flat plate shapes [14, 15, 23]. This treatment simplifies the research, but it ignores that many winged diaspores have heteromorphic wings [18].

*Firmiana* Marisili is a small genus of the expanded Malvaceae family that includes 16 species distributed in Africa and Asia [24]. The plants in this genus produce a unique aggregate fruit during sexual reproduction, which contains seeds that continue to be connected to the spoon-shaped pericarp after maturity [25, 26]. When the petiole of the fruitlet breaks, seeds leave the maternal plant and are dispersed by wind, together with the pericarp [27]. *F. simplex* (Linnaeus) W. Wight, also called the phoenix tree in Chinese legend, which states that the Phoenix bird will only perch on *F. simplex* plants, is a medium-sized tree widely distributed in Asia, Europe, and temperate regions of the USA. Although some authors have reported that the fruit of this plant spins in the air after detaching from plants, similar to the winged seeds of conifers and the samaras of Acer plants [14, 27], they did not consider that its fruit has a different morphology than those winged seeds or samaras and that this morphology could influence the seed dispersal process. Here, studying the fruit morphology and seed dispersal of *F. simplex*, we aimed to determine the following: 1. If there any significant differences in morphology between the fruit of *F. simplex* and other winged diaspores; 2. How the morphology of the fruit is formed in *F. simplex*; 3. What effects these fruit morphological characteristics have on seed dispersal.

## Results

### Fruit development

**Fig. 1:**
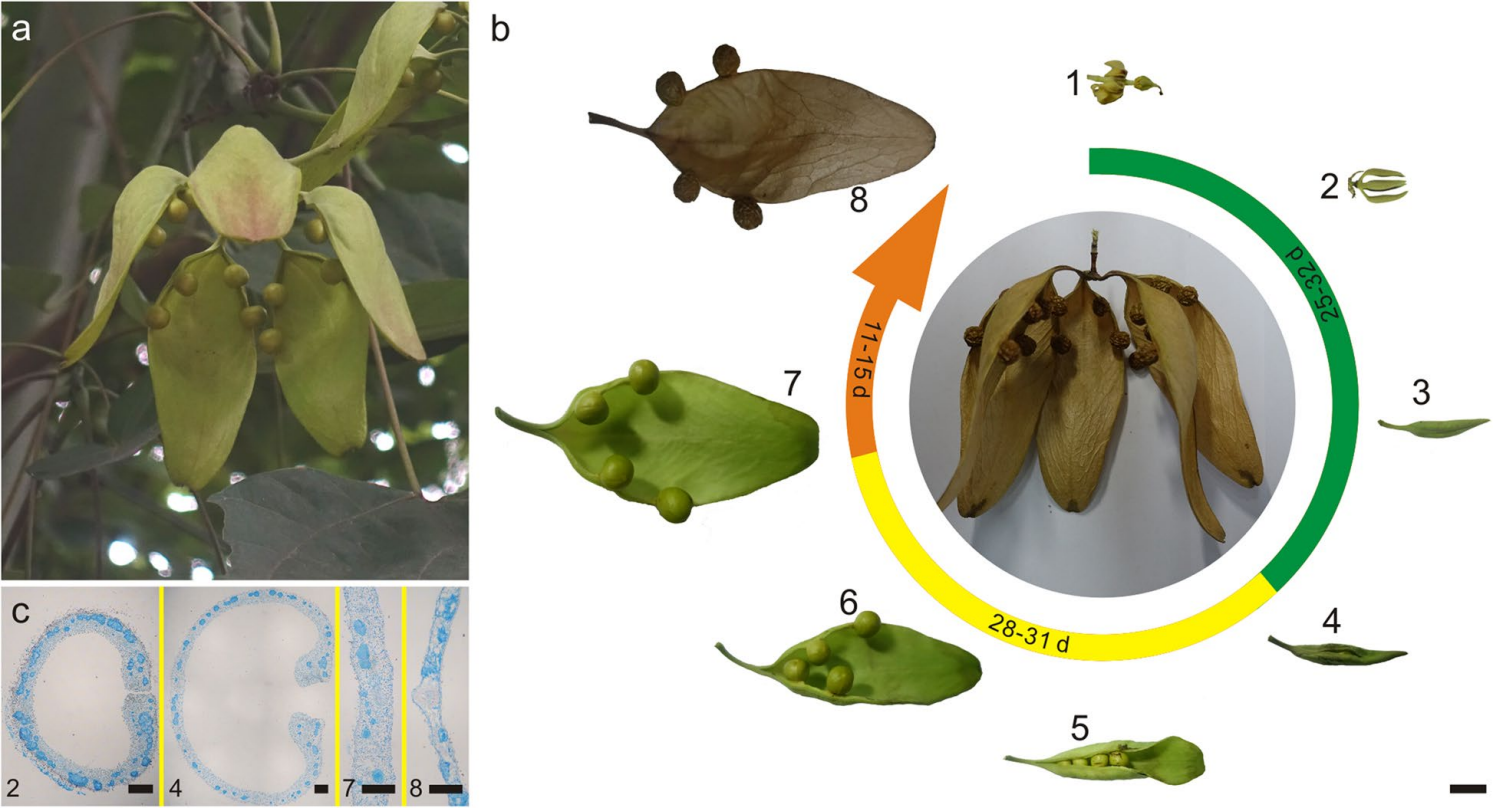
Development of the fruit of *Firmiana simplex*. **a** An immature fruit developing on the tree. **b** The morphology of the fruitlet at different developmental stages from flowering to fruit ripening. The middle inset shows a mature aggregate fruit. The coloured curves indicate the different stages of fruit development: green, before fruit cracking; yellow, fruit cracking and expanding; orange, fruit drying. The values on the curves indicate the duration of each stage. **c** The internal structure of pericarps at several different stages of fruit development. The numbers correspond to different developmental stages in (**b**)

*F. simplex* required approximately two-to-three months from pistil fertilization to fruit maturation during which time the fruits were consistently hanging on the fruit branches (Fig. 1a). The fruit of *F. simplex* is characterized as aggregate follicle, and each fruitlet is completely developed from the carpel of the gynoecium. Each gynoecium of the female flower contained five carpels, which separated in the ovary region but fused in the style area in anthesis (Fig. 1b1). Once the petals dropped, all gynoecium fruitlets were completed separated, leaving only basic connections to the fruit stalk (Fig. 1b2). The closed ovary area expanded rapidly with the elongation of fruitlet (7–10 days; Fig. 1b3). Many glandular hair structures were distributed on the inner wall of the ovary, through which a large amount of liquid was secreted (Fig. 1c). After 11–15 days, the abdominal suture of the fruitlet slow began to crack from the ovary area, and the liquid in the ovary disappeared (Fig. 1b4 and 1c). When the entire abdominal suture was cracked, the tip of the fruitlet unfolded into a sheet and bent to the back, while the pericarp in the ovary area was still sealed and the seeds were not fully exposed (Fig. 1b5). In the following 6–8 days, the peel unfolded slowly until the fruit was fully open, at which time seeds could be observed growing alternately on either side of the pericarp (Fig. 1b6). As a result, the pericarp expanded and deformed to the same shape as the mature fruit, While the pericarp and seed color remained green (Fig. 1b7). Tissue sections revealed that the pericarp contained many plump parenchyma cells (Fig. 1c). In the final period of development, the color of the fruit changed from green to light red, then yellow, and finally to brown (Fig. 1b8). The water content of fruit, as well as the fruit mass, decreased continuously. In the mature fruit, all parenchyma in the pericarp was destroyed, and only a large number of cavities and scattered vascular bundles remained (Fig. 1c).

### Fruit morphology

**Fig. 2:**
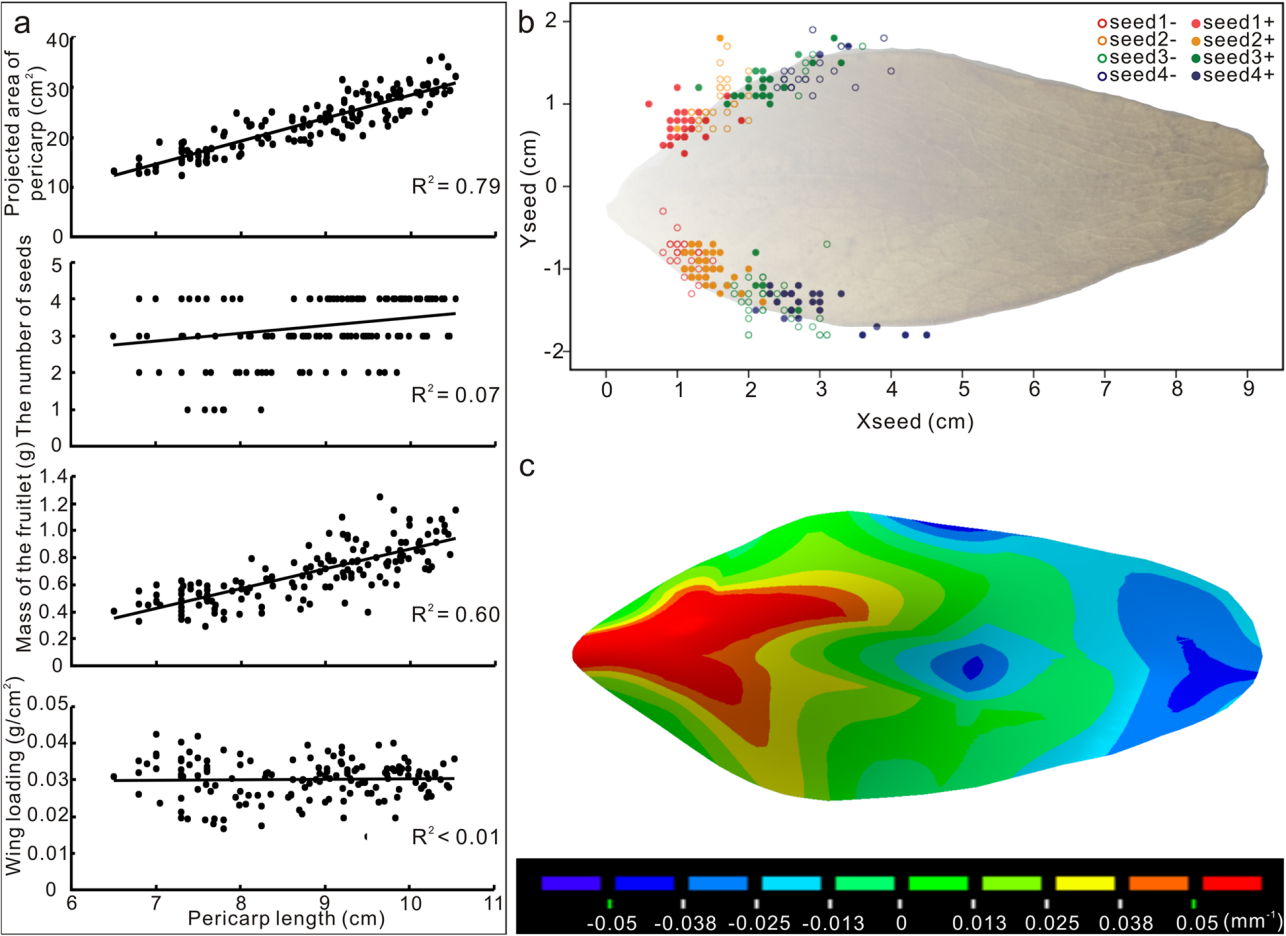
Morphological characteristics of the fruit of *Firmiana simplex*. **a** Pericarp projected area, seed number, total mass, and wing loading of the fruit with different lengths. Scattered points represent different fruit samples in each graph, and the straight lines are the linear fitting curves of the data. **b** The distribution of seed positions on the pericarp. Coloured circles represent the different seeds in the fruit. Hollow circles and solid circles correspond to seeds starting from the left and right sides of the pericarp along the long axis, respectively. **c** The curvature distribution of the pericarp surface. The ventral surface of the pericarp points inward

The fruitlet was the dispersal units used by plants of *F. simplex* due to the fragile fruitlet petiole in the mature fruit. In the fruitlet, the pericarp was 8.7 ± 1.1 cm (mean ± s.d. *N* = 150) in length, 3.5 ± 0.6 cm in width, 22.6 ± 5.6 cm^2^ in projected area, 3.2 ± 0.5 mm in thickness, and 0.22 ± 0.08 g in mass. As the pericarp length increased, the projected area of the pericarp grew significantly (Fig. 2a). The number of seeds ranged from 1 to 4 (*N* = 150), and the total seed mass in the fruitlet was 0.46 ± 0.14 g. There was a linear relationship between fruitlet mass and peel length, but a linear relationship between wing loading and peel length was not identified (Fig. 2a). Regardless of the number of seeds in the fruitlet, the seeds generally grew alternately on either side of the pericarp along the length axis. All seeds were distributed in the area near the base, which was no more than half the length of the pericarp (Fig. 2b).

Although there may be considerable differences in pericarp size, seeds number, and the distribution of seeds in the fruitlet among different plants, fruits of the same plant, or even different fruitlets, fruitlets generally have similar shapes. The curvature analysis results of the pericarp surface showed that the average curvature of the basal area had different signs from that of the region near the top (Fig. 2c). The basal area formed a convex surface on the backside of the fruitlet, and the top area formed a concave surface on the dorsal side. The middle area of the pericarp was relatively flat (Fig. 2c). Comparing the distribution of seeds on the pericarp and its curvature distribution of the pericarp, the seeds were observed to be distributed on the relatively flat edge of the convex basal area.

### Flight performance of fruit

**Fig. 3:**
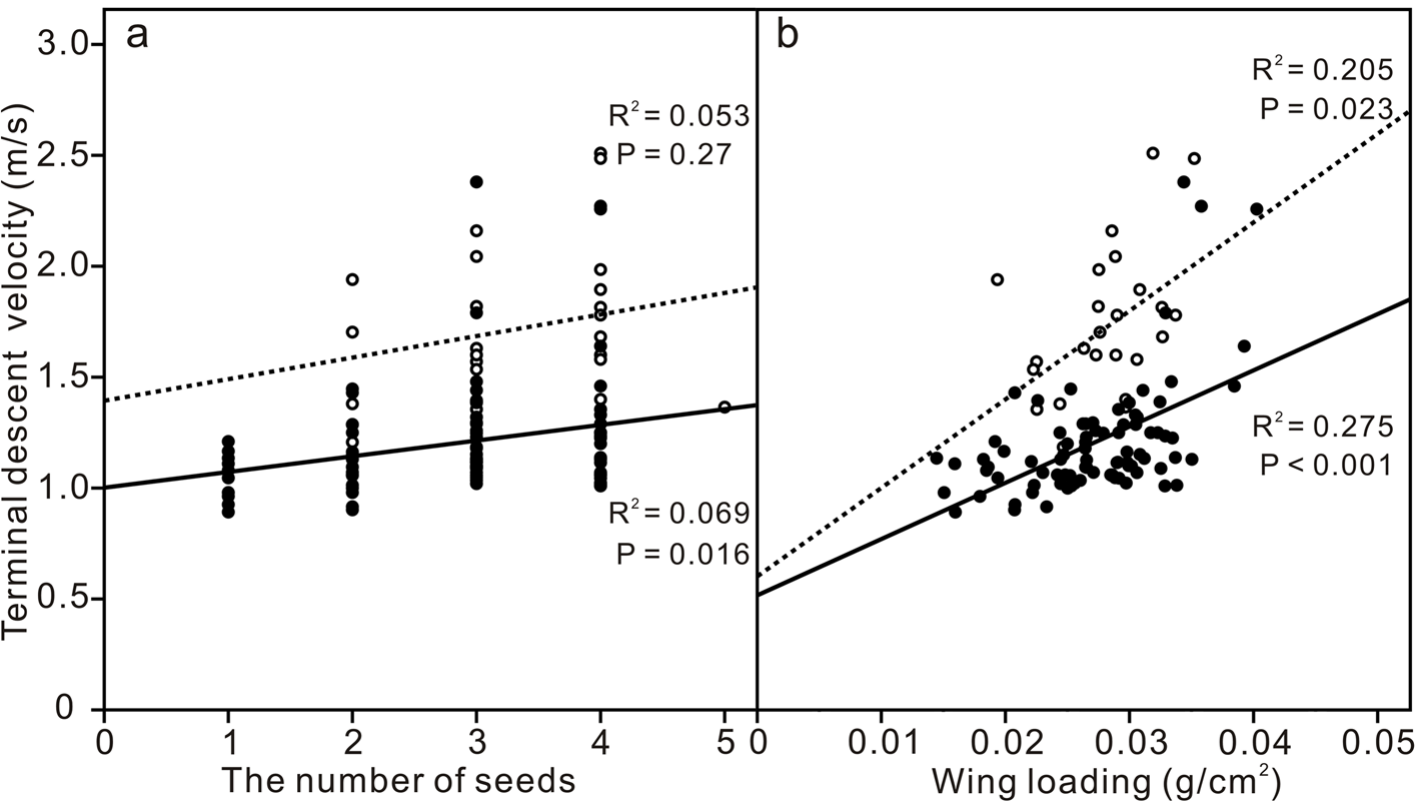
Terminal descent velocity versus number of seeds (**a**) and wing loading (**b**) in *Firmiana simplex*. Solid points and hollow points represent two groups of fruit spinning with their dorsal and ventral sides downward, respectively, and the solid lines and dotted lines are the linear fitting curves of these two groups of data, respectively

In the drop test, most mature fruits (109/120) could enter a stable spinning state after falling 1–2 m from the starting position. The fruitlets of *F. simplex* showed two orientations during descent after becoming stable spinning. The majority of the fruitlets (*N* = 84) were ventral-side up, while a few fruitlets (*N* = 25) were dorsal-side up. The terminal velocity, spin frequency and coning angle of the ventral-side up fruitlet were 1.20 ± 0.27 m/s, 10.57 ± 2.15 Hz and 22.62 ± 9.05 deg., respectively. The dorsal-side up fruitlets returned 1.72 ± 0.34 m/s, 8.66 ± 2.26 Hz, and 28.44 ± 14.49 deg. for the same factors, respectively. There were significant differences in terminal velocity and spin frequency (*P* < 0.01) between the two orientations, but there was no significant difference in coning angle (*P* > 0.05). The linear regression results showed no clear linear relationship between the terminal velocity and the number of seeds of a fruit. Still, there was a weak linear relationship between terminal velocity and wing loading, which was more pronounced in the ventral-side up fruit (Fig. 3).

In the controlled drop test, except for the Group 0 fruit without seeds, most of the fruits of the other four groups produced stable spinning. During spinning, there were significant differences in terminal velocity (F_3, 68_ = 6.02, *P* < 0.01) and spin frequency (F_3, 68_ = 22.14, *P* < 0.01) among the four groups with different seed counts, but there was no significant difference in coning angle between these groups (F_3, 68_ = 2.56, *P* = 0.06). Numerically, the terminal velocity was correlated with wing loading. In the three groups of fruits with different seed distributions (Groups 1, 2, and 3), completely different flight performances between the groups could be observed (Table 1). One fruit in Group 1 showed spinning with its dorsal side up, while two fruits in Group 2 failed to spin.

**Table 1:**
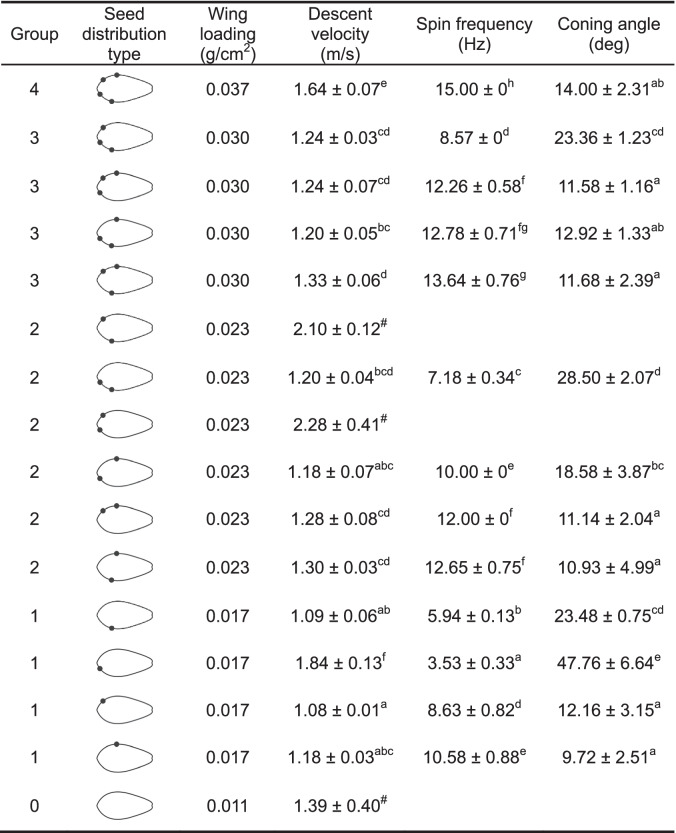
Flight performances of *Firmiana simplex* fruits with different seed distributions in the controlled drop tests

Values with different superscript letters differ significantly at *p* < 0.05 in analysis of variance (ANOVA). The superscript # represents no spinning during fruit falling

### Falling process of fruit models with different shapes

**Fig. 4:**
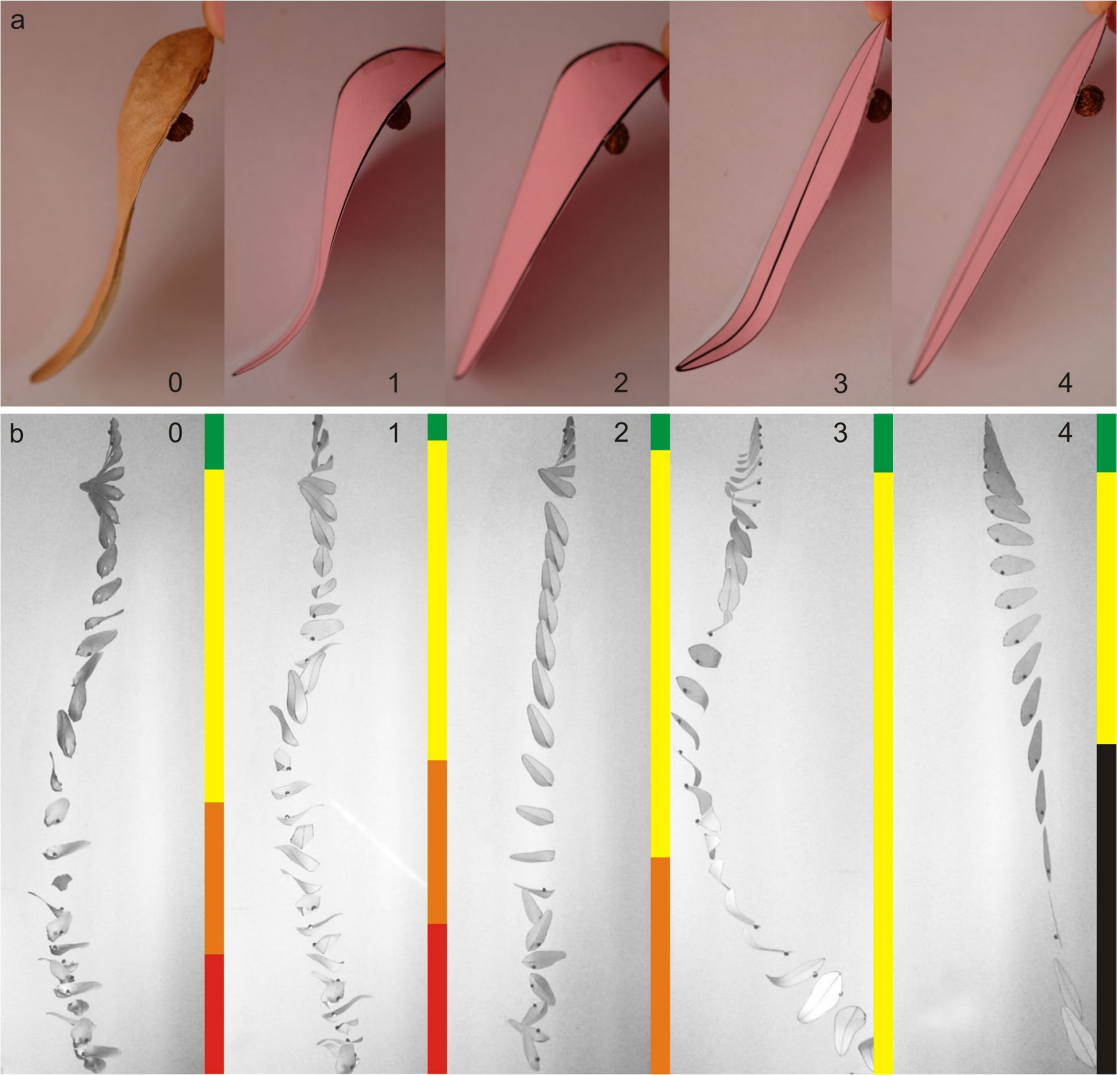
Flight-attitude changes of fruit during falling in *Firmiana simplex*. **a** Experimental samples of actual fruit and paper models containing only one seed per fruit. **b** Posture changes of experimental samples during falling. The coloured bars represent different postures: green, downward acceleration; yellow, tumbling; orange, decelerated spinning; red, stable spinning; black, falling in a near straight line. The number 0 identifies the actual fruit and other sequential numbers identify different paper models: 1, Model I, in which the surface curvature of all areas were similar to the actual pericarp; 2, Model II, in which the curvature of the ovary area was similar to the actual pericarp but the top area was flat; 3, Model III, in which the curvature of the top was similar to the actual pericarp but the ovary area was flat; 4, Model IV, in which all areas were flat

The projected area of all paper models of *F. simplex* fruit was similar to that of the actual, but the surface curvature distribution of the three models differed substantially (Fig. 4a). The fruit of *F. simplex* underwent several stages from the stationary position to stable spinning during the falling process: an acceleration stage, whereby the fruit began to fall, accelerate from the stationary point, and maintain a similar posture; a rolling stage, in which the fruit rolled around different axes in the air and then fell at an accelerated pace; and a spinning deceleration stage, whereby the fruit began to rotate approximately around the vertical axis, and the descent velocity decreased slowly. Subsequent to these stages, with the fruit began to spin stably at a relatively constant frequency and a low descent velocity (Fig. 4b0). The falling path of the fruit was close to vertical in a windless environment. Model I, which had the same curvature distribution as the actual fruit, showed a falling process similar to the actual, but differing in the duration of each stage (Fig. 4b1). Model II, which a concave ovary and flat tip showed three stages in the descent phase. The fruit in the first two stages was similar to the actual fruit, but it rotated around the changing axis in the third stage, and the descent velocity was not significantly reduced (Fig. 4b2). Model III had a curved tip and a flat ovary. After a short period of slow acceleration in the falling process, this model rolled continuously until it reached the ground. The falling path remained an irregular curve even in a windless environment (Fig. 4b3). During descent, Model IV underwent two similar stages to Model III, but after the ovary area turned downward, the model fell at high speed, and the falling path approximated a straight line (Fig. 4b4).

### Aerodynamics of fruit models with different shapes

**Fig. 5:**
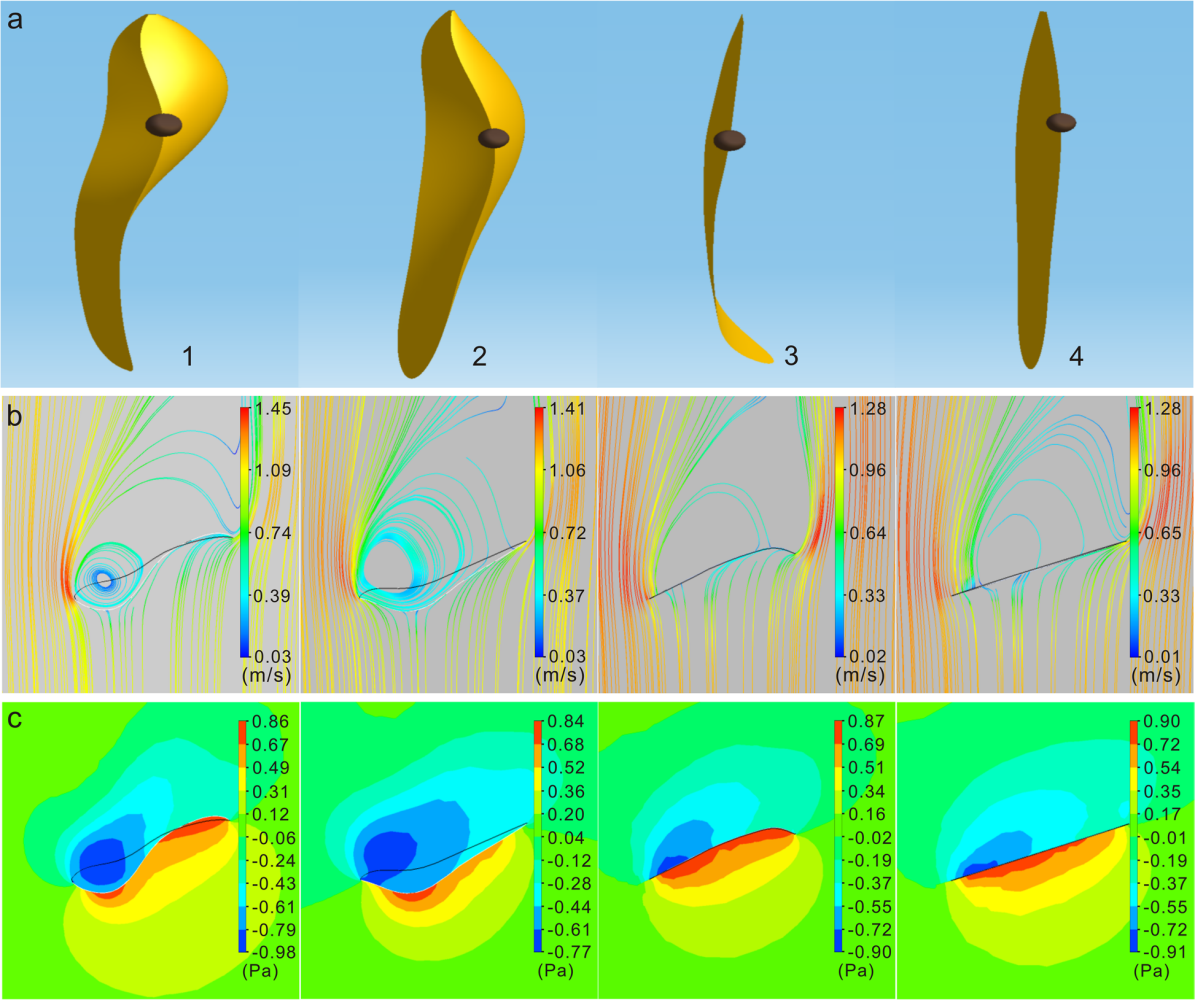
Results of aerodynamics simulation of a static pericarp in *Firmiana simplex*. **a** 3D models of pericarps in digital simulation. The sequential numbers identify the pericarp corresponding to the shape of different paper models. **b** Streamline distributions in the long axis section of pericarp models. **c** Pressure distributions on the long axis section of pericarp models

The 3D digital models of the fruit had similar curvature distributions to the four paper models (Fig. 5a). When the angle between the length axis of all models and the air flow direction was consistent with the coning angle of the actual spinning fruit, the streamlines on the dorsal side of all models bypassed the pericarp from the edge and formed a vortex near the ovary on the ventral side (Fig. 5b). The streamlines of the vortex in Models III and IV were relatively looser than those of Models I and II. The pressure distribution near the models revealed a dorsal high-pressure area and a ventral low-pressure region in all models (Fig. 5c). The pressure distribution of Model I was similar to that of Model II. There was a low-pressure center on the ventral side and an extreme high-pressure distribution on the dorsal side of the ovary area. However, the extreme high-pressure distribution of Model I was occurred in the tip area, while there was no extreme high-pressure distribution in the tip area of Model II. The pressure distribution of Model III was similar to that of Model IV. Extreme low pressure was distributed in the ventral ovary area and extreme high pressure approximated an even distribution along the length axis on the dorsal side.

## Discussion

Wind dispersed seeds generally have cilia or wing-like appendages [7, 11]. They are classified as either single-winged or multi-winged diaspores depending on the number of wings attached to the seeds [12, 28]. *F. simplex* fruits are single-winged diaspores, similar to seeds of the cone plant and the fruits of Acer [13, 15]. Although similar to those plants, the fruit of *F. simplex* has a thin wing, representing a dried pericarp, which has a surface that is no longer flat. The wing has a large curvature in its base and tip area, resembling a spoon. The shape of this "spoon" is close to symmetrical on both sides along the length axis, while the wing of the samara of other groups generally does not have bilateral symmetry, which means that the center of mass of the fruit is on one side of the length axis [14, 17, 21]. The seeds of *F. simplex* are alternately borne on two sides of the pericarp base so that the center of mass of the fruit is located on one side of the fruit is near the pericarp base. This distribution characteristic of the center of mass is common in single-winged diaspores in which the seeds to stabilize their spinning in the process of falling [17, 21].

Most spinning winged diaspores contain only a single seed, usually located at one end of the wing [12, 14]. As a diaspore that can spin stably during falling, the fruit of *F. simplex* can produce multiple seeds, and the number of seeds per fruit varies. The seed mass accounts for a high proportion in the fruit mass; therefore, the total mass of the fruit has a relatively large range. However, due to the positive correlation between wing size and fruit mass, the variation range of fruit load is limited. In the falling process, the terminal velocity of fruit, like that of other winged diaspores, increases mainly with the increase of wing loading [6, 7], and has no clear relationship with the size of the fruit and the number of seeds. The terminal velocity directly impacts the dispersal distance of the winged diaspore [28, 29], where this distance does not appear to be correlated with the seed number contained in the fruits. This differs from the situation with *Lonchocarpus pentaphyllus*, a leguminous plant with 0–3 seeds per fruit, whereby the fruit of that plant that contains multiple seeds tends to fall closer to the maternal plant after being dispersed by the wind than the fruit with a single seed [30].

The seed implantation position on the pericarp affects the distributions in the fruit, which could completely alter the dispersal pattern. The seed distribution determines the center of mass of fruit, which can impact the falling process and prevent stable spinning in certain cases. In nature, seeds within the fruit of *F. simplex* have relatively fixed positions that alternate on either side of the pericarp base, ensuring that the center of mass of the fruit is limited to a relatively small area and the fruits are able to descend at a low velocity by spinning.

Although there can be considerable differences in size, mass, wing loading, number of seeds, and seed distribution among fruits of *F. simplex*, the spoon shape of their wings, the pericarps, are similar. According to the results of our drop test of the paper models, this shape is essential for the stable spinning of the fruit. In other plants with single-winged diaspores, when seeds are spinning stably during the falling process, drag and lift forces on the flat wing occur simultaneously, and are balanced with the weight of the fruit [7, 17, 18]. The lift is mainly provided by the leading-edge vortex (LEV) on the upper edge of the wing, and the action center of drag force is separated from the fruit centroid [15, 21]. During stable spinning of the fruit of *F. simplex*, there should be a similar mechanical equilibrium. However, because seeds in the fruit grow in the basal ovary area of the pericarp rather than one end, this area accounts for a large proportion of the fruit, making the centroid of the entire fruit close to the geometric center of the pericarp. If the pericarp of *F. simplex* had a flat shape, the action center of the drag force on the fruit would be close to the centroid, and the fruit would fail to stabilize its spinning during the descent [17]. To overcome this, *F. simplex* uses a concave shape in the base area of the pericarp, which ensures that the air drag mainly affect the pericarp tip and not the concave base. The curved shape of the pericarp tip is also necessary to stabilize spinning. Similar to the family Dipterocarpaceae, the curved pericarp tip of *F. simplex* increases the resultant forces of lift and drag that are balanced with gravity [18, 31].

Different morphology of winged diaspores usually relates to the seeds or fruit development process [13, 32, 33]. The fruitlets of *F. simplex* are follicles and are developed entirely from the carpel. The wing, which is the pericarp, is formed from the entire ovary wall, unlike the samaras of Acer plants, whose wings are composed of only a part of the ovary wall [34]. Similar to the development of other follicles, the fruitlet of *F. simplex* has obvious dorsal bulging during development, and which a concave area at the base after fruit ripening [35]. However, unlike other follicles, the fruitlet of *F. simplex* dehisces long before ripening [36, 37]. The originally enclosed spindle-shaped pericarp unfolds into a wing in subsequent development. During this period, there is sufficient time for the pericarp tip to complete the reverse bending and create the spoon-shape of the fruitlet. During development, the fruit changes shape and show clear differences in internal structure at different development stages. Before dehiscence, the closed ovary of the fruitlet is filled with a large amount of liquid, which may protect or provide nutrition for the seeds. When the fruit is ready to mature, the green wet pericarp and seeds quickly lose water and become dry, resulting in a significant decline in the mass of the entire fruit.

The carpel of *F. simplex* in anthesis contains 1 to 4 ovules, of which only some will become plump seeds after fruit development [25]. Therefore, the number of seeds in the mature fruit is indefinite, as the distribution position of these seeds on the pericarp. This can also be attributed to the fruit's special carpel structure and development process. In the carpel of *F. simplex*, two lateral vascular bundles usually extend alternately into the ovules along the abdominal suture [36]. Although all ovules in the carpel appear to be attached to the middle abdominal suture, they are closer to one of two edges of the ovary wall. When the pericarp subsequently dehisces, the two edges move to opposite sides with the ovules closely related. The seeds are alternately distributed on both sides of the wing in the fruit.

There is a close relationship between the total seed mass and pericarp morphology during fruit development of *F. simplex*. An increase in total seed mass usually leads to a corresponding increase in pericarp size. The expansion of the pericarp area originates from the growth and division of pericarp cells [37]. Many hormones are required to promote these changes in the cells. Hormones during fruit development mainly come from seeds, and an increase in seed number may promote the growth and division of pericarp cells [38]. Correspondingly, because the pericarp, which is green during most of fruit development, can conduct photosynthesis, a larger pericarp can provide more or heavier seeds with the nutrients needed for development. Nevertheless, the growth of the pericarp as a wing may also require substantial resources from the maternal plant [13, 39]. Having multiple seeds attached to the same wing ensures the effective use of these resources for plants that dispersed seeds by wind [30]. The terminal velocity of *F. simplex* diaspores when falling is not increased with greater seed numbers. Therefore, its fruitlet with multiple seeds does not lose long-distance dispersal ability. Many other plants that disperse seeds by wind tend to contain only one seed in a diaspore [30], whereas the multiple seeds per diaspore in *F. simplex* create more adaptability and may be favored by natural selection. However, some carpels in the gynoecium of *F. simplex* only contain 1–2 ovules, and several seeds in fruit can be destroyed by external factors during fruit development; therefore, some mature fruits may only contain 1–2 seeds [25, 36].

The early dehiscence of pericarp during fruit development has an important adaptive significance in the evolution of *Firmiana*. This early dehiscence helps the pericarp develop into a spoon-shaped wing, and the unfolded shape after dehiscence can increase the efficiency of pericarp photosynthesis in supplying resources for fruit development. However, the premature dehiscence of the pericarp is not common in angiosperms [36, 37]. The closed pericarp before fruit ripening plays an important role in protecting internal seeds from external factors [37, 40]. The premature dehiscence of the pericarp greatly increases the risk of internal seeds being destroyed and may reduce the survival rate of angiosperm offspring. This also applies to the fruits of *Firmiana*. However, in *Firmiana*, the premature dehiscence of the pericarp does not significantly impact the survival of the progenies. Some species in this genus have become invasive plants due to their strong sexual reproduction [24]. This may indicate that the diaspore produced by this unique development process provides a benefit to the survival and reproduction of plants through efficient wind dispersal that is greater than the loss caused by premature dehiscence of the pericarp.

## Conclusions

The early cracking of the ventral carpel suture during *F. simplex* fruit development causes the mature fruit to have a unique curved shape and seed distribution pattern. These characteristics allow the fruit of *F. simplex* to spin stably in the air, and its seeds can be dispersed by wind after separation from the mother plant. However, this differs from the morphology of other winged seeds or samaras. These findings deepen our understanding of the wind-dispersed mechanism of seeds and may provide a good model for further study on the relationship between fruit development and seed dispersal.

## Methods

### Plant materials and development observation

Nineteen plants of *Firmiana simplex* were used for this study, which were 8.2 to 15.7 m in height and grew near Sand Lake (30°57′ N, 114°33′ E), Wuhan, Hubei Province, China. Several pistils of *F. simplex* were collected every 10 days from fertilizing female flowers to fruit full maturity. The morphology of pistils in different developmental stages was observed under a stereo-microscope (Olympus SZ-61, Tokyo, Japan). To investigate the changes in the internal structure of the pericarp during fruit development, paraffin sections were performed on fruits at different developmental stages. Twenty samples of pistils after pollination, pistils about to crack, green unfolded fruits, and fully mature fruits were fixed in formalin-acetic acid-alcohol (FAA; 5: 5: 90, v/v). The ovary parts of these fixed pistils were then cut at a thickness of 10 μm through the standard steps of paraffin sectioning, stained with 0.5% toluidine blue, and observed in a bright field under an optical microscope (Zeiss Axiolab, Jena, Germany). All observational experiments were conducted from June to October in both 2018 and 2019.

### Morphological measurement

A total of 150 mature fruitlets from 10 different natural plants were collected. The length and width of these fruits were measured using electronic calipers. The pericarp projected area and seed distribution in each fruit were measured using the following methodology. First, the fruit was placed on a white viscose-covered table with the ventral side of the fruit facing upward and photographs were taken using a digital camera approximately 20 cm above the table. Second, the photographs were transferred to a computer, and measurement data was obtained using the ImageJ software (Version 1.44). For the measurement of seed distribution, the intersection of the pericarp and petiole was designated the origin, and the line of the midrib of the pericarp lies was set as the x-axis. The mass of each part of the fruit, including the pericarp and all seeds, was weighed on an analytical balance (Yueping FA2204B, Shanghai, China). Finally, 10 pericarps were randomly selected from all collected fruits, and scanned completely using a laser 3D scanner to obtain 3D files of the pericarp surface (3Doe LDS, Shenzhen, China). The 3D files were imported into CATIA V5 (Dassault, Paris, France) to analyze the curvature distribution of the pericarp surface.

### Drop test

A hundred and twenty mature fruitlets were randomly collected from ten plants grown in the field. The length, pericarp projection area, and total mass of these fruits were measured according to the same method as that of the previous section. The drop test was then performed in a so-called “dead air space” [41]. Each fruit was dropped twice from a height of 3 m in quiescent air, and the falling process over 1 m was recorded at a rate of 60 fps using a digital camera (FUJI XT10, Tokyo, Japan). The descent videos were transferred to a computer to obtain the frame sequence of the falling process of each fruit with Adobe Photoshop (CS 5, Adobe Systems Inc, San Jose, USA). The flight characteristics of the fruit, including descent velocity, rotation frequency, and coning angle, were then analysed according to a similar video analysis method described by Nathan et al. [42].

The number and distribution of seeds on the pericarp can impact the flight process of fruits. To test this potential impact, a control experiment was performed. A fruit with medium length, medium projected area, and four seeds of similar size was selected from the fruits collected in the previous experiment. All seeds in the fruit were removed, and different numbers of seeds were pasted on the pericarp in different distribution patterns with small pieces of the dual adhesive tape, to form 16 different modified fruits. According to the number of seeds pasted, these modified fruits were divided into five groups. The flight characteristics of all modified fruit were measured according to the same method as the previous drop tests, and the measurements were repeated five times for each fruit.

To determine the influence of pericarp shape on the flight process of the fruit, a set of deformed fruit models were constructed in a similar way to that of Stevenson et al. [22]. Two natural fruits with approximately the average length and projected area were selected from previously collected samples, and all seeds on the fruit were stripped. One pericarp was reconnected to a peeled seed at its original position by using a small piece of double-sided tape, and used as a natural control fruit. The other pericarp served as the shape template for all paper models. The four pericarp paper models were obtained by cutting, scanning, laser cutting, and pasting using red tissue paper with an area density similar to the actual pericarp (approximately 70 g/m^2^) (see additional file 1). The four paper models corresponded to pericarps with different curvature distributions: Model I, the surface curvatures of all areas were similar to that of the actual pericarp; Model II, the curvature of the ovary area was similar to the actual pericarp, but the top area was flat; Model III, the curvature of the top was similar to the actual pericarp but the ovary area was flat; and Model IV, all areas were flat. All paper models were pasted with a seed previously removed from the template fruit to obtain four deformed fruit models, and the pasted position was consistent with the projection position of the seed pasted on the natural control fruit. The four fruit models and the control fruit were dropped five times, with zero initial velocity from a height of 3 m in quiescent air, and the falling process of the first two meters at a rate of 500 fps, was recorded with a high-speed camera (SONY DSC-RX 100 V, Tokyo, Japan) at a distance of 1.5 m. These high-speed videos were used to obtain the flight attitude diagram during the falling process using Adobe Photoshop software. The time interval of adjacent flight attitudes was selected as 30 ms. In addition, by counting the number of attitudes in these diagrams, the mean descent speed of samples in the initial 2 m descent was calculated.

### Digital simulation

The aerodynamic mechanism causing the fruit shape to influence the flight process was complex, but it could be briefly analysed by a computational fluid dynamics (CFD) approach. Referring to the 3D scanning data of the actual pericarp, four 3D digital models of the fruit corresponding to the paper models in the drop test were created using CATIA software. These models were imported into CFD simulations through ANSYS 14.5 software (ANSYS Inc., Canonsburg, USA) for aerodynamics analysis. In all simulations, the air velocity was set to 1 m/s, which is similar to the terminal descent velocity of natural fruit in the static air, and the angle between the basal apical axis of the pericarp model and the airflow was equal to the remainder of the mean coning angles of fruit spinning from our experiments. More details of 3D model creations and the CFD simulations were provided in Additional file 2. The flow-field for the entire fluid domain was obtained for each simulation. The velocity field and pressure distribution on the length symmetry planes of each model were compared to determine the aerodynamic differences between the fruits with different shapes in the assumed spin state.

### Data analysis

The relationships between fruit length and the other morphological variables, including peel projected area, seed number, total fruit mass, and wing loading, were analyzed by fitting linear regressions. ANOVA tests were performed on all variables of flight performance among different groups of fruits in the controlled drop test to determine the effect of different seed numbers and seed distributions on the seed dispersal process.

## Supplementary Information

**Additional file 1.** Manufacturing method of paper models of Firmiana simplex fruit. 

**Additional file 2.** Details of aerodynamic simulations with four digital models of Firmiana simplex fruit.

**Additional file 3.** The raw data of all statistical analysis in the article.

## Data Availability

The data generated or analyzed in this study are included in this article and its supplementary information files. Voucher specimens in our study are stored in the herbarium of Hubei University (EU), and the specimen numbers are E190037-E190051 (identified by Wen-Long Fu). Other materials that support the findings of this study are available from the corresponding author on reasonable request.
