# Supplementary material for: “Phoenix in Flight”: an unique fruit morphology ensures wind dispersal of seeds of the phoenix tree (Firmiana simplex (L.) W. Wight)
Source: BMC Plant Biol. 2022 Mar 12;22:113. doi: 10.1186/s12870-022-03494-z (PMC8917737; doi:10.1186/s12870-022-03494-z)
Supplement: Supplementary file 1 — Additional file 1. Manufacturing method of paper models of Firmiana simplex fruit. [file 12870_2022_3494_MOESM1_ESM.pdf]

Manufacturing method of paper models of *Firmiana simplex* fruit:

To make paper models of the fruit, a series of operations were performed: First, the projected image of the template pericarp is recorded using a digital camera. Second, it is cut with anatomical scissors from the petiole to about half the length of the pericarp along the midvein, flattened and scanned in a laser scanner (HP M1005). Third, the two pericarp images before and after cutting were imported into a computer, resized to be close to the actual pericarp using Adobe Photoshop software, and then two contour lines that are smooth, symmetrical and close to the shape of the template pericarp were redrawn respectively (Fig. S1a). The areas enclosed by the two contour lines were respectively called shape 4 and shape 1. Shape 1 was compressed to 0.9 times the original length along its axis of symmetry to obtain shape 2, while shape 4 was stretched to 1.1 times the original length along its axis of symmetry to obtain shape 3. All four shapes were used to laser cut the base shape of the models from tissue paper with an area density similar to that of actual pericarp (70 g/m^2). The dotted line areas of the paper pieces with shape 1 and shape 2 were bonded together with double-sided adhesive to form a concave curved surface. The tip of the paper with shape 1 was curled to the bending degree close to the actual peel tip to obtain model I, the tip of the paper with shape 2 was kept straight to obtain model II, the tip of the paper with shape 3 was also curled to the bending degree close to the actual peel tip to obtain model III, and the paper with shape 4 kept the whole surface straight to obtain model IV (Fig. S1b).

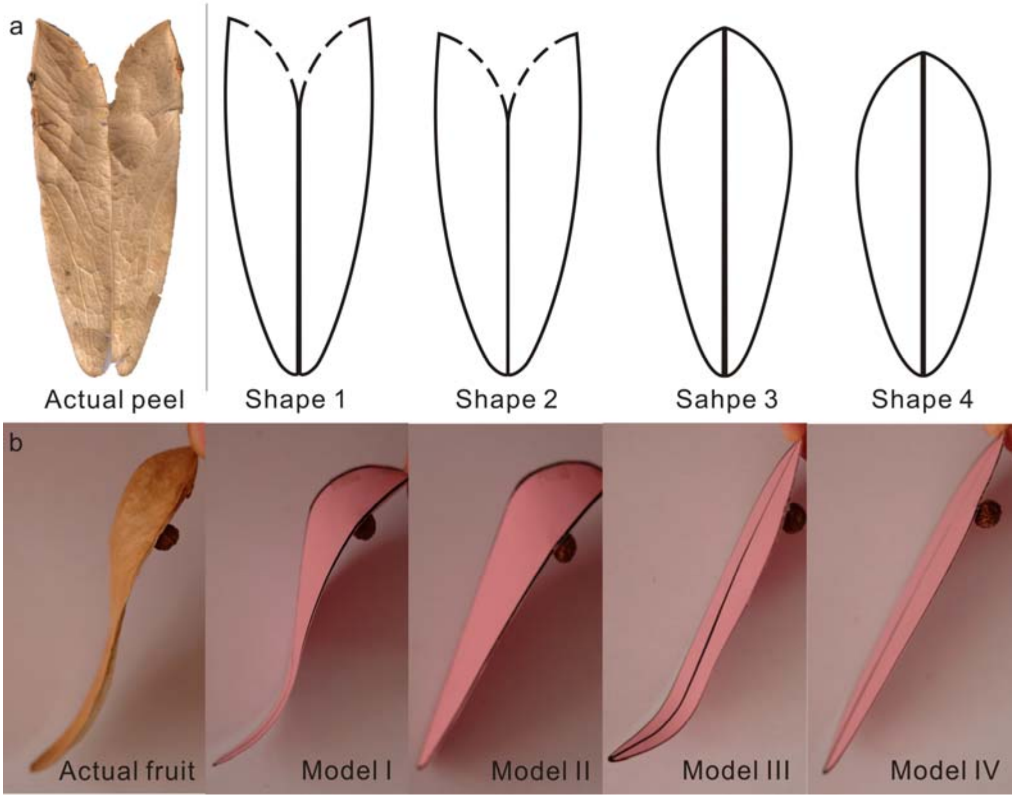

Fig. S1. The two-dimensional outlines (a) and three-dimensional shapes (b) of the actual fruit and fruit paper models of *Firmiana simplex*. The solid lines in (a) are the outlines of the peel paper models, and the dotted lines are the bonding lines of these paper models.
