# Supplementary material for: “Phoenix in Flight”: an unique fruit morphology ensures wind dispersal of seeds of the phoenix tree (Firmiana simplex (L.) W. Wight)
Source: BMC Plant Biol. 2022 Mar 12;22:113. doi: 10.1186/s12870-022-03494-z (PMC8917737; doi:10.1186/s12870-022-03494-z)
Supplement: Supplementary file 2 — Additional file 2. Details of aerodynamic simulations with four digital models of Firmiana simplex fruit. [file 12870_2022_3494_MOESM2_ESM.pdf]

Details of aerodynamic simulations with four digital models of *Firmiana simplex* fruit:

Both groups of peel models were analyzed using the same digital wind tunnel. The digital wind tunnel was cylindrical structure with diameter of 0.5 m and height of 2.0 m, the models were located in the central area of the wind tunnel, one end of the cylindrical wind tunnel was set to the air inlet, the other end was set to the air outlet, and the cylindrical surface was set to the "wall". The angle between the long axis of all models and the direction of air flow were set to 68 deg. (remainder of the coning angle of *F. simplex* fruit during spinning in the air), and the velocity of air flow was set as 1.0 m/s (approximate to the average terminal velocity of the *F. simplex* fruit obtained in drop test) and the outlet pressure was set as 0 Pa. The air streamline near each peel model and the pressure distribution around the model were analyzed.
